# The functional connectivity of language network across the life span: Disentangling the effects of typical aging from Alzheimer’s disease

**DOI:** 10.3389/fnagi.2022.959405

**Published:** 2022-09-23

**Authors:** Marie Rafiq, Mélanie Jucla, Laura Guerrier, Patrice Péran, Jérémie Pariente, Aurélie Pistono

**Affiliations:** ^1^Department of Neurology, Neuroscience Centre, Toulouse University Hospital, Toulouse, France; ^2^Toulouse NeuroImaging Center, Toulouse University, Inserm, University Paul Sabatier (UPS), Toulouse, France; ^3^Octogone-Lordat Interdisciplinary Research Unit (EA 4156), University of Toulouse II-Jean Jaurès, Toulouse, France; ^4^Department of Experimental Psychology, Ghent University, Ghent, Belgium

**Keywords:** language, language network, functional connectivity, aging, Alzheimer’s disease

## Abstract

Language is usually characterized as the most preserved cognitive function during typical aging. Several neuroimaging studies have shown that healthy aging is characterized by inter-network compensation which correlates with better language performance. On the contrary, language deficits occur early in the course of Alzheimer’s disease (AD). Therefore, this study compares young participants, healthy older participants, and prodromal AD participants, to characterize functional connectivity changes in language due to healthy aging or prodromal AD. We first compared measures of integrated local correlations (ILCs) and fractional amplitude of low-frequency oscillations (fALFFs) in language areas. We showed that both groups of older adults had lower connectivity values within frontal language-related areas. In the healthy older group, higher integrated local correlation (ILC) and fALFF values in frontal areas were positively correlated with fluency and naming tasks. We then performed seed-based analyses for more precise discrimination between healthy aging and prodromal AD. Healthy older adults showed no functional alterations at a seed-based level when the seed area was not or only slightly impaired compared to the young adults [i.e., inferior frontal gyrus (IFG)], while prodromal AD participants also showed decreased connectivity at a seed-based level. On the contrary, when the seed area was similarly impaired in healthy older adults and prodromal AD participants on ILC and fALFF measures, their connectivity maps were also similar during seed-to-voxel analyses [i.e., superior frontal gyrus (SFG)]. Current results show that functional connectivity measures at a voxel level (ILC and fALFF) are already impacted in healthy aging. These findings imply that the functional compensations observed in healthy aging depend on the functional integrity of brain areas at a voxel level.

## Introduction

### Language in healthy aging

Language is usually characterized as the most preserved cognitive function during typical aging (in particular in terms of verbal knowledge: Park et al., 2002). However, several aspects of language production show an age-related decline. For example, older participants have lower capacities than young participants during production tasks (e.g., verbal fluency: Meinzer et al., 2009; semantic association: Pistono et al., 2019a) and they generally experience more tip-of-the-tongue states (Burke et al., 2018). The observation of age-related increases in task-based brain functional activation led to the hypothesis that the preservation of cognitive function is underpinned by compensatory mechanisms. Several theories [e.g., HAROLD (Cabeza, 2002); PASA (Davis et al., 2009); STAC (Reuter-Lorenz and Park, 2014)] have been formulated to explain these compensatory mechanisms.

Few studies have looked at functional networks rather than discrete brain regions to assess age-related changes and compensation. Resting-state functional connectivity is one of the current methods to describe the relationship between neural activation patterns of anatomically separated brain regions, which is consistent with the notion of networks used to perform a specific cognitive task. The use of task-free functional magnetic resonance imaging (fMRI) to study the aging brain allows the avoidance of potential confounds and limitations encountered in language production paradigms, such as head motion, performance anxiety, trial exclusion, etc. To reveal language functional networks, several studies have used a seed-based approach, using a key language area, such as the left inferior frontal gyrus (IFG) or the left superior temporal gyrus, as a seed. Gertel et al. (2020), for example, found that older adults have stronger functional connectivity between the left IFG and right hemisphere executive function regions, associated with improved behavioral pedrformance on the Stroop task. Similarly, a previous study by our group (Pistono et al., 2021a) showed that older adults have increased connectivity between the language network and the multiple-demand (executive) network. The strength of connectivity between these two networks was positively correlated with fluency performance in the older group. Seed-based analyses (using the IFG as a seed) showed similar results. In fact, older adults had stronger connectivity between the seed and areas that are not part of the language network, such as the precuneus, which was correlated with better language performance. Therefore, it would appear that older adults rely more on regions outside the language network to maintain a good level of language performance. Besides long-range connectivity, Pistono and colleagues also took account of measures of local connectivity, through integrated local correlation (ILC) in core regions of the language network. This measure determines the “local coherence” of functional networks. It is characterized by the strength and sign of connectivity between a given voxel and the neighboring areas within a region of interest. This team showed that local connectivity is reduced within the language network in typical aging, the left superior frontal gyrus (SFG) being the most weakened area. Therefore, it is possible that the increased functional connectivity noted with other networks in the older group is due to local coherence alterations in several areas of the language network. This also implies that local measures could be more sensitive to the effects of healthy aging.

### Language in Alzheimer’s disease

Unlike typical aging, language deficits occur early in the course of Alzheimer’s disease (AD). Most studies have shown impairment in fluency tasks and confrontation naming tasks, usually attributed to lexical-semantic impairment (Taler and Phillips, 2008). Many authors have investigated lexical-semantic impairment during discourse processing and found an increase in word-finding difficulties (e.g., Ash et al., 2007; de Lira et al., 2011). Regarding resting-state functional connectivity, only few studies have focused on language networks in AD, reporting lower functional connectivity in AD compared to healthy controls (Weiler et al., 2014; Mascali et al., 2018; Montembeault et al., 2019). However, connectivity changes were not, or were only marginally correlated with AD participants’ language performance. At a prodromal stage, functional connectivity changes seem quite limited. In fact, Pistono et al. (2021b) found no inter-group differences between prodromal AD participants and healthy controls during seed-based analyses, neither using the left frontal inferior gyrus nor the left superior temporal gyrus as a seed. Nonetheless, the use of machine-learning techniques allowed the discrimination of healthy controls and patients with prodromal AD using atrophy patterns in language-related areas or functional connectivity patterns between language areas. In particular, patients with prodromal AD had increased connectivity between three key language areas, correlated with better language performance (Pistono et al., 2021b). Therefore, at a prodromal stage, patients can make use of compensatory mechanisms despite important gray matter loss.

Other compensations are probably at stake at this stage. Integrated local connectivity, for example, has proven to be a marker of both alteration and compensation in AD. Several studies (see Liu et al., 2008 for a review) have indicated that patients with AD have increased local coherence in several areas (e.g., bilateral cuneus), which could compensate for alterations in other areas (e.g., posterior cingulate cortex/precuneus, Liu et al., 2008). Other measures seem to follow a similar pattern, such as the fractional amplitude of low-frequency oscillations (fALFFs). fALFF measures the power within a specific frequency range divided by the total power in the entire detectable frequency range. Zeng et al. (2019), for example, showed that patients with prodromal had decreased fALFF activity in several areas (e.g., precuneus) but increased activity in other areas (e.g., IFG). Therefore, AD seems characterized by both functional connectivity impairment and compensation, but measures of local connectivity and fALFF have never been more specifically performed in the language network. Additionally, a global interpretation of current literature is hardly possible given the heterogeneity of measures, clinical criteria, or stage of AD. In particular, the lack of significant differences between healthy older adults and participants with prodromal AD could be due to impairments of healthy aging or could reflect compensations that occur in healthy aging and persist at a prodromal stage of AD. Therefore, this study aims to improve the discrimination of functional connectivity changes due to healthy aging from those due to prodromal AD.

### Current study

On the one hand, previous literature has shown that healthy aging is characterized by local connectivity impairment within the language network and inter-network compensation that correlates with better language performance. On the other hand, few studies have analyzed the language functional network in AD. Some have shown decreased connectivity in the language network (e.g., Weiler et al., 2014), while others have shown that language performance also relies on functional compensation [i.e., increased connectivity between specific areas as shown by Pistono et al. (2021b)]. It also remains to be known whether AD participants differ from healthy controls on measures related to specific regions of interest (i.e., local correlations and fALFF in each language-related area). Therefore, this study compared young participants, healthy older participants, and participants with prodromal AD to characterize language functional connectivity changes due to healthy aging or AD.

First, we considered measures of integrated local correlations (ILCs) and fALFF in each area of a language network (using a functional atlas, Fedorenko et al., 2010). Pistono et al. (2021a) showed that local correlation in several language-related areas was impaired in healthy aging. Therefore, we assumed that this measure is a hallmark of aging, and not specific to AD. Consequently, we predicted that both healthy older adults and prodromal AD participants have decreased values in language-related areas compared to young adults. We also measured fALFF to study language specifically, which has never been done. We expected a similar pattern as ILCs.

Second, as mentioned above, healthy aging is also characterized by increased inter-network connectivity, which is usually interpreted as a compensatory mechanism. To further investigate this assumption, we used a seed-based approach based on areas that best discriminate young adults from healthy aging and patients with prodromal AD (i.e., on local correlations and fALFF measures). More precisely, we predicted that discriminant areas (i.e., areas for which healthy older adults and prodromal AD participants differ from young adults) had more connections with non-language areas in healthy older adults than young adults during seed-based analyses. On the contrary, prodromal AD participants did not present such compensations.

Additionally, we performed correlations between connectivity changes and language performance. We predicted that higher local coherence and fALFF are correlated with better language performance in healthy aging, as well as connectivity with areas outside the language network. This provided additional support in favor of compensatory mechanisms in healthy aging. In other words, this study aimed to show that local changes in the functional network of language characterize aging. However, healthy older adults are able to compensate for such alterations with increased inter-network connectivity, while prodromal AD participants are not.

## Materials and methods

### Participants

This study was approved by the ethics committee (IDRCB: 2015-A01416-43). Participant recruitment followed the same procedure as that described by Pistono et al. (2021a). Participants were right-handed and native French speakers. All the participants provided written, informed consent before participating in the study and received monetary compensation for their participation.

Participants with prodromal AD were selected if they presented with a memory complaint and had no concomitant history of neurological or psychiatric disease other than AD. They underwent the following pre-inclusion assessment:

- Autonomy in daily living [Instrumental Activities of Daily Living (IADL, Graf, 2008)] and [Clinical Dementia Rating (CDR, Morris, 1993)];

- Global cognition [Mini-Mental State Evaluation (MMSE, Folstein et al., 1975)];

- Anterograde verbal memory [Free and Cued Selective Reminding Test (FCSRT, Van der Linden et al., 2004)]. The FCSRT was selected because it is based on semantic cueing that allowed us to control for effective registration of the list of words and to facilitate the retrieval of stored information. The FCSRT was administered according to the procedure described by Sarazin et al. (2007).

- Amyloid assessment with cerebrospinal fluid (CSF) analysis by lumbar puncture: CSF biomarker levels of total tau (T-Tau), phospho-tau (P-Tau), Aβ42, and Aβ40 were measured using an ELISA method (Innogenetics, Ghent, Belgium). Innotest Amyloid Tau Index (IATI) was calculated. P-Tau ≥ 60 pg/ml and IATI ≤ 0.8 were deemed to be suggestive of AD. In case of an ambiguous profile (P-Tau < 60 pg/ml or IATI > 0.8), we calculated the Aβ42/Aβ40 ratio and a score <0.045 was considered to be compatible with a diagnosis of AD.

Individuals with typical AD were included at the prodromal stage, which corresponds with the following criteria: MMSE ≥ 24; CDR ≤ 0.5, and based on the IWG-2 (i.e., International Working Group 2) criteria: evidence of a gradual and progressive change in memory function reported by patient or informant for more than 6 months and demonstrated by an episodic memory test, and CSF evidence of AD.

Healthy control participants underwent the same pre-inclusion neuropsychological assessment as the prodromal AD group. They were included if they had no memory complaints and no history of neurological or psychiatric disease and an MMSE ≥ 27. They were excluded if they presented with cognitive impairment (test scores <–1.5 SDs) during the pre- or post-inclusion neuropsychological assessment.

### Cognitive and clinical evaluation

All participants underwent a comprehensive neuropsychological assessment. Visual recognition memory was assessed with the Doors and People test (Baddeley et al., 1994). Short-term memory and working memory were evaluated with the WAIS-III Digit Span and Backward Digit Span subtest (Wechsler, 1997). Cognitive flexibility was assessed with the Trail Making Test (TMT, Reitan, 1958). Praxis was explored with Mahieux’s assessment (Mahieux-Laurent et al., 2008), and gnosis with the Visual Gnosis Evaluation Protocol (VGEP, Agniel et al., 1992). Apathy and depression were also measured, using the Starkstein scale (Starkstein et al., 1992) and the Beck Depression Inventory (Beck et al., 1961) to exclude participants with severe symptoms.

Language was assessed with the GREMOTs assessment (Bézy et al., 2016). The GREMOTs is a computerized language battery dedicated to early-stage neurodegenerative diseases. This battery evaluates both oral and written language, production, and comprehension at different levels. Phonological processing is assessed through non-word repetition, non-word reading, and non-word writing under dictation. Lexical processing covers naming tasks (objects, actions, famous faces), fluency tasks [semantic (fruit), grammatical (verbs), phonemic (V)], word repetition, word reading, word writing under dictation, and oral and written semantic verification. Syntactic processing covers sentence production, sentence writing under dictation, sentence repetition, order execution, and syntactic comprehension. Discourse processing is based on spontaneous language, interview, narrative discourse, and oral and written discourse comprehension. It takes approximately 2 h to complete.

### Behavioral analyses

Kruskal–Wallis or Chi-square test was used to measure sociodemographic matching. Comparisons regarding the cognitive evaluation were made using the Kruskal–Wallis test. The results were corrected for multiple comparisons according to Bonferroni’s method. Statistical analysis was done using the R software version 4.0.2 (R Core Team, 2018) and Jamovi software (Love et al., 2021).

### MRI acquisition

MRI scans were performed for all participants using a 3-T scanner (Philips Achieva dStream, Philips Healthcare, Best, Netherlands). A 3D-T1 image was acquired for anatomical reference with the following parameters: TR = 8 ms, TE = 3.7 ms, flip angle = 8°°, matrix size = 256 × 256 mm, 170slices, and voxel size (in mm) = 0.9 × 0.9 × 1. Whole-brain resting-state fMRI images were obtained with the following parameters: TR = 2,837 ms, TE = 40 ms, flip angle = 90° 46 interleaved acquisition, slice thickness = 3 mm, matrix size = 80 × 80 mm, 200 volumes, and total scan time 10 min. During scanning, participants were instructed to keep their eyes closed but to stay awake and avoid thinking of anything in particular. All participants affirmed that they were fully awake during the 10 min of scanning.

### Preprocessing

The data were analyzed using the Conn toolbox (Version 19c), implemented in MATLAB. The preprocessing pipeline of the functional images included: functional realignment and unwarp, slice-timing correction, outlier identification, normalization to the montreal neurological institute (MNI) template, and smoothing with a Gaussian kernel of 8 mm. This step created a scrubbing covariate (containing the potential outliers scans for each participant) and a realignment covariate (containing the six head motion parameters). The six head motion parameters plus their associated first-order derivatives, the identified outlier scans, white matter, and CSF signals and the effect of rest were then removed by the CompCor method. The resulting preprocessed images were band-pass filtered (0.01–0.1 Hz) to remove physiological high- and low-frequency noise (e.g., cardiac and respiratory fluctuations).

### Functional connectivity

#### Definition of language-related areas

The 13 regions of interest (ROIs) from the original parcels of Fedorenko et al. (2010) were chosen. This set of language-sensitive regions has been defined and validated with a localizer task designed to target brain regions sensitive to word- and sentence-level meaning. This includes 10 ROIs within the left hemisphere: IFG, IFG-orbitofrontal gyrus (IFG-Orb), middle frontal gyrus, SFG, anterior temporal gyrus, middle-anterior temporal gyrus, middle-posterior temporal gyrus, posterior temporal gyrus, angular gyrus, and cerebellum; three ROIs in the right hemisphere: middle-anterior temporal gyrus, middle-superior temporal gyrus, and cerebellum.

#### Integrated local correlations

Local correlation maps were measured based on Pearson’s correlation coefficient of the time courses between the current and neighboring voxels (ILC, Deshpande et al., 2009), using the Conn Toolbox. An 8-mm spatial convolution kernel was used for ILC bounds. ILC was determined specifically in language-related areas. Inter-group comparisons of mean ILC values from the 13 ROIs were performed using one-way analysis of variance (ANOVA). Bonferroni corrections for multiple comparisons were applied.

#### Fractional amplitude of low-frequency oscillations

Fractional amplitude of low-frequency oscillation measures the relative contribution of spontaneous low-frequency oscillations within a specific frequency band to the entire detectable frequency range. It is calculated as a relative measure of BOLD signal power within the frequency band compared to that over the entire frequency spectrum. It represents the ratio of root mean square of the BOLD signal at each individual voxel after vs. before low- or band-pass filtering (Zou et al., 2008). Similar to the measures of local correlations, we measured mean fALFF in each language-related areas.

#### Seed-based analyses

Seeds were defined based on results from ILC and fALFF analyses (i.e., areas that significantly differed between the two groups). Correlation maps were constructed by correlating the averaged BOLD signal dynamic of the region of interest with the BOLD signal of every other single voxel. To enforce a Gaussian distribution of the correlation data, Pearson’s correlation coefficients were then transformed to z-scores using the Fisher r to z transformation for subsequent *t*-tests. These individual *z*-value maps were entered into an one−sample *t*−test to determine the functional network correlated with spontaneous activity of the seed region within each group [*p* < 0.05 FWE (family-wise error) corrected at the cluster level, with a voxel wise *p* < 0.001]. We then performed two−sample *t*−tests to detect inter-group differences due to aging (i.e., young adults vs. healthy older adults) and AD status (i.e., healthy older adults vs. participants with prodromal AD). The threshold for second-level maps was set at *p* < 0.05 FWE at the cluster level; *p* < 0.001 at the voxel level.

#### Correlations with language performance

Intragroup correlations were performed with language tasks that were known to be sensitive to the effects of typical aging or early AD and regions with significant group differences in functional connectivity. Therefore, we selected lexical tasks as they are both sensitive to the effect of healthy aging (e.g., Meinzer et al., 2009; Vogel-Eyny et al., 2016) and AD (e.g., Clark et al., 2009; Pistono et al., 2019b): semantic fluency (total number of words for the category “fruit”), phonemic fluency (total number of words starting with the letter V), grammatical fluency (total number of words with the category verb), object naming, and famous faces naming. Bonferroni–Holm corrections for multiple comparisons were applied.

## Results

### Participants

Twenty-three healthy young adults (28 ± 4 years old), 24 healthy older adults (70 ± 4 years old), and 24 participants with prodromal AD (73 ± 7 years old) were recruited. The three groups were matched for gender (young group: 12 women; healthy older group: 13 women; prodromal AD group: 11 women) and level of education (see Table 1).

**TABLE 1:** Pre-inclusion assessment.

	Healthy young adults	Healthy older adults	Participants with prodromal AD	*p*-value	Pairwise comparisons
Age	28 ± 4	70 ± 4	73 ± 7	**<0.001**	Young adults < Healthy older adults < prodromal AD
Level of education	14 ± 2	12 ± 4	12 ± 4	0.15	–
MMSE	28.7 ± 1	29 ± 1	25.5 ± 2.6	**<0.001**	Young adults = Healthy older adults > prodromal AD
FCSRT	38.6 ± 3.8	32.29 ± 4.79	14.17 ± 9.69	**<0.001**	Young adults > Healthy older adults Young adults > prodromal AD Healthy older adults > prodromal AD

Results represent mean ± SD. Results that are significant after Bonferroni–Holm corrections are in bold.

There was a main effect of MMSE [*X*^2^(2) = 46.7, *p* < 0.001]: healthy young adults and healthy older adults had higher MMSE than prodromal AD participants. There was also a main effect of FCSRT [*X*^2^(2) = 31.9, *p* < 0.001].

### Language

Overall, the young and older groups performed better than the prodromal AD group on all the tasks, namely, lexical, syntactic, and phonological processing. However, the older group performed better than the young group for naming famous faces and spelling words (Table 2).

**TABLE 2:** Performance on the language assessment (Kruskal–Wallis).

		Healthy young adults	Healthy older adults	Participants with prodromal AD	*p*-value	Pairwise comparisons
**Lexical processing**	Repetition, words (/10)	9.91 ± 0.29	9.38 ± 1.01	9.17 ± 1.05	**0.005**	Young adults = Healthy older adults > prodromal AD
	Grammatical fluency (category: verbs)	33.60 ± 11.80	35.21 ± 11.66	27.08 ± 11.23	0.05	–
	Semantic fluency (category: fruits)	21.90 ± 4.27	19.33 ± 3.38	15.04 ± 6.03	**<0.001**	Young adults = Healthy older adults > prodromal AD
	Phonemic fluency (letter V)	20.40 ± 6.28	17.29 ± 6.12	17 ± 8.01	ns	–
	Object naming (/36)	34.6 ± 0.99	34.7 ± 1.40	32.63 ± 1.91	**<0.001**	Young adults = Healthy older adults > prodromal AD
	Action naming (/36)	33.70 ± 1.97	33.13 ± 3.25	31.13 ± 2.8	**0.001**	Young adults = Healthy older adults > prodromal AD
	Famous face naming (/10)	6.48 ± 1.93	8.75 ± 1.15	4.83 ± 2.78	**<0.001**	Healthy older adults > Young adults > prodromal AD
	Reading, words (/30)	29.70 ± 0.47	29.71 ± 0.55	29.33 ± 0.92	ns	–
	Spelling, words (/12)	10.80 ± 1.13	11.58 ± 0.504	10.04 ± 1.33	**<0.001**	Healthy older adults > Young adults > prodromal AD
	Oral semantic verification (/18)	17.20 ± 0.85	17.04 ± 1.27	15.96 ± 1.6	**0.004**	Young adults = Healthy older adults > prodromal AD
	Written semantic verification (/18)	16.70 ± 1.11	16.3 ± 1.69	14 ± 2.21	**<0.001**	Young adults = Healthy older adults > prodromal AD
**Syntactic processing**	Repetition, sentences (/4)	3.78 ± 0.52	3.46 ± 0.78	3.42 ± 0.65	ns	–
	Order execution (/6)	5.96 ± 0.21	5.96 ± 0.20	5.79 ± 0.42	ns	–
	Sentence production (/6)	5.96 ± 0.21	5.75 ± 0.68	5.25 ± 0.94	**0.002**	Young adults = Healthy older adults > prodromal AD
	Syntactic comprehension (/24)	22.80 ± 2.33	21.25 ± 2.51	18.92 ± 3.62	**<0.001**	Young adults = Healthy older adults > prodromal AD
	Text comprehension (time in seconds)	39.10 ± 7.54	49.3 ± 15.73	80.88 ± 30.51	**<0.001**	Young adults = Healthy older adults > prodromal AD
**Phonological processing**	Repetitions, non-words (/6)	5.87 ± 0.34	5.54 ± 0.66	5.08 ± 0.93	**0.002**	Young adults = Healthy older adults > prodromal AD
	Reading, non-words (/15)	14.30 ± 0.83	14.67 ± 0.64	13.79 ± 1.06	**0.002**	Young adults = Healthy older adults > prodromal AD
	Spelling, non-words (/6)	5.48 ± 0.51	5.50 ± 0.59	4.96 ± 1.04	ns	–

Results represent mean ± SD. Results that are significant after Bonferroni–Holm corrections are in bold.

### Other cognitive evaluation

The prodromal AD group had a significant alteration in visual recognition memory, short-term memory, processing speed, inhibition, and visual gnosis than both the young and the healthy older group. They were also more apathetic, as shown with the Starkstein test. The healthy older group was slower than the young group during TMT A and B (Table 3).

**TABLE 3:** Performance on the neuropsychological assessment (Kruskal–Wallis).

	Healthy young adults	Healthy older adults	Participants with prodromal AD	*p*-value	Pairwise comparisons
Doors and people test, set A	11.30 ± 1.11	10.78 ± 1.38	8.00 ± 2.55	**<0.001**	Young adults = Healthy older adults > prodromal AD
Digit span forward	6.30 ± 1.15	6.00 ± 1.00	5.21 ± 0.98	**<0.001**	Young adults = Healthy older adults > prodromal AD
Digit span backward	5.13 ± 1.39	4.83 ± 1.40	4.04 ± 0.91	0.02	–
Trail making test, A	22.10 ± 8.52	38.79 ± 12.50	51 ± 16.41	**<0.001**	Young adults > Healthy older adults > prodromal AD
Trail making test, B-A	29.40 ± 12.10	55.13 ± 27.86	114.22 ± 81.83	**<0.001**	Young adults > Healthy older adults > prodromal AD
Praxis	23.00 ± 0.21	22.60 ± 0.78	22.00 ± 1.41	0.004	–
VGEP	35.80 ± 0.52	35.26 ± 1.10	33.79 ± 2.87	**<0.001**	Young adults = Healthy older adults > prodromal AD
Beck	2.26 ± 2.47	2.58 ± 2.21	3.29 ± 3.28	ns	–
Starkstein	6.96 ± 3.17	9.50 ± 4.19	11.78 ± 4.60	**0.001**	Young adults = Healthy older adults > prodromal AD

Results represent mean ± SD. Results that were significant after the Bonferroni–Holm correction is in bold.

### Functional connectivity: local correlations

**FIGURE 1:**
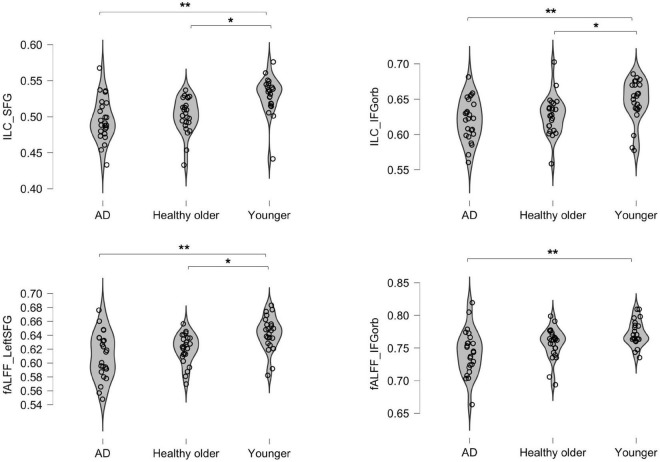
Inter-group differences in integrated local correlation (ILC) and fALFF values. The *y* axis represents individual ILC and Fractional amplitude of low-frequency oscillation (fALFF) values. *p < 0.05; **p < 0.001.

Three areas significantly differed between groups: the left IFG; left SFG; and left IFG-Orb. As shown in Table 3 and Figure 1, healthy older adults did not significantly differ from young adults for IFG values and did not differ from prodromal AD participants for SFG and IFG-Orb values. Results from areas that were not significant after correction for multiple comparisons are detailed in Supplementary material 1.

### Functional connectivity: Fractional amplitude of low-frequency oscillation

Two areas significantly differed between groups: the left SFG and left IFG-Orb. As shown in Table 4 and Figure 1, healthy older adults did not significantly differ from young adults for IFG-Orb values, and neither did they differ from prodromal AD participants for SFG values. Results that were not significant after correction for multiple comparisons are detailed in Supplementary material 2.

**TABLE 4:** Inter-group differences for integrated local correlation (ILC) and Fractional amplitude of low-frequency oscillation (fALFF).

	Young adults	Healthy older adults	Prodromal AD	*p*-value	Pairwise comparisons
ILC-IFG	0.63 ± 0.02	0.61 ± 0.03	0.60 ± 0.03	<0.05	Young adults = Healthy older adults > prodromal AD
ILC-SFG	0.53 ± 0.03	0.50 ± 0.03	0.50 ± 0.03	<0.001	Young adults > Healthy older adults = prodromal AD
ILC-IFG-Orb	0.65 ± 0.03	0.63 ± 0.03	0.62 ± 0.03	<0.01	Young adults > Healthy older adults = prodromal AD
fALFF-SFG	0.65 ± 0.03	0.62 ± 0.02	0.61 ± 0.03	<0.001	Young adults > Healthy older adults = prodromal AD
fALFF-IFG-Orb	0.77 ± 0.02	0.76 ± 0.02	0.74 ± 0.03	<0.01	Young adults > prodromal AD Healthy older adults = Young adults; Healthy older adults = prodromal AD

Results represent mean ± SD.

### Correlations with language performance

In the young group and the group with prodromal AD, there were no correlations between ILC, fALFF, and language performance. In the healthy older group, grammatical fluency score was positively correlated with mean ILC values in SFG (*p* < 0.01, *r* = 0.55) and IFG-Orb (*p* < 0.05, *r* = 0.50). The naming of famous faces was positively correlated with mean fALFF values in SFG (*p* < 0.01, *r* = 0.54). There were also positive correlations between the naming of famous faces and mean fALFF values in IFG-Orb (*p* < 0.05, *r* = 0.42), as well as mean ILC values in SFG (*p* < 0.05, *r* = 0.45), but these two correlations were not significant after correction for multiple comparisons.

### Seed-based analyses

As the SFG and IFG-Orb were the most discriminant regions both during ILC and fALFF measures, they were selected as seeds to perform further analyses of the functional language network.

#### Seed superior frontal gyrus

Regions positively and negatively correlated with the SFG in each group are detailed in Supplementary material 3.

**FIGURE 2:**
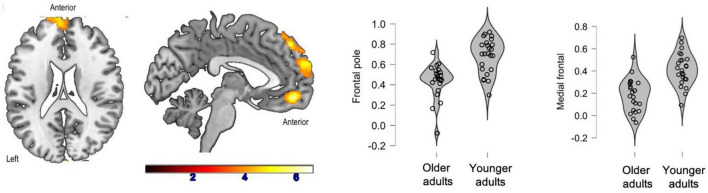
Significant clusters (in yellow) after inter-group comparisons (young adults vs. healthy older adults). The violin plots show connectivity values for each participant in each group.

There were no inter-group differences between healthy older adults and prodromal AD participants. Two clusters were significantly more correlated with the SFG in the young adult group compared to the healthy older adult group: the left and right frontal poles as well as the medial frontal cortex (Figure 2).

Additionally, connectivity between the SFG and the frontal poles was positively correlated with phonemic fluency (*p* < 0.01, *r* = 0.57) and naming of famous faces (*p* < 0.01, *r* = 0.54) in healthy older adults.

#### Seed inferior frontal gyrus-orbitofrontal gyrus

Regions positively and negatively correlated with the IFG-Orb in each group are detailed in Supplementary material 4.

**FIGURE 3:**
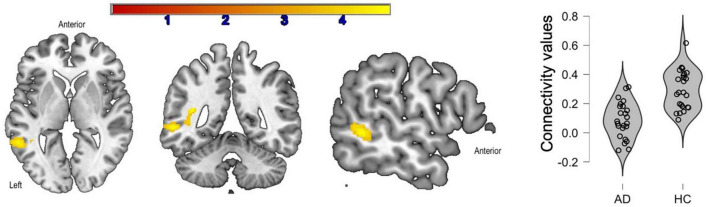
Significant clusters (in yellow) after inter-group comparisons [healthy older adults (HC) vs. prodromal Alzheimer’s disease (AD)]. The violin plots show connectivity values for each participant in each group.

There were no inter-group differences between young adults and healthy older adults. Compared to prodromal AD participants, healthy older adults had greater connectivity between the IFG-Orb and the middle temporal gyrus (Figure 3). In the healthy older group, no correlations were found with language performance. In the group with prodromal AD, the connectivity between the seed and the middle temporal gyrus was positively correlated with phonemic fluency, although these correlations were not significant after correction for multiple comparisons (*p* < 0.05, *r* = 0.37).

## Discussion

This study compared young participants, healthy older participants, and participants with prodromal AD in order to characterize functional connectivity changes in language due to healthy aging or AD. It showed that, on a behavioral level, healthy older adults had similar or better performance compared to young adults, while participants with prodromal AD had significant impairment. Nonetheless, both groups of older adults showed functional changes: lower connectivity values within several language-related areas, as well as decreased whole-brain connectivity.

The language assessment showed that healthy older adults do not significantly differ from young adults, and performed even better on spelling tasks and naming of famous faces. On the contrary, the group with prodromal AD had lower performance on each language production level: lexical processing, as well as syntactic processing and phonological processing. Lexical-semantic impairment is consistent with previous literature (Joubert et al., 2010; Barbeau et al., 2012) but not all studies showed syntactic or phonological impairment in early AD. Kemper et al. (1993) found preservation of syntactic abilities in AD using written sentence analyses, while studies based on syntactic comprehension (similar to one of the tasks we used) have shown significant impairment, starting at the prodromal stage (Lambon Ralph et al., 2003). This suggests that this task is more sensitive to AD than sentence production. In terms of phonological processing, AD participants’ capacities are usually described as being preserved during both oral and written tasks (Taler and Phillips, 2008). Current results can also be explained by the use of a language battery specifically dedicated to neurodegenerative disorders at a prodromal stage (i.e., rather than tests derived from vascular aphasia batteries).

Regarding functional connectivity analyses, measures that focus on specific areas of interest underlined the effect of age. In fact, both healthy older adults and participants with prodromal AD had lower ILC and fALFF values compared to young adults in several language-related areas: SFG, IFG, and IFG-Orb. Therefore, both groups of older adults exhibited a local functional decrease, which is in line with our predictions. Additionally, current results show that healthy older adults do not differ from participants with prodromal AD on most ILC and fALFF measures. Consequently, it would appear that functional connectivity measures at a voxel level within language ROIs are sensitive to the effect of aging (i.e., both healthy and pathological aging). Significant areas are frontal ROIs, the SFG being the most significant one. These findings are consistent with the fact that the prefrontal cortex is the region most disrupted by healthy aging (Zanto and Gazzaley, 2019).

However, seed-based analyses could better discriminate healthy aging from prodromal AD. For this analysis, the most significant areas at the voxel level were chosen as seeds. The left SFG was the most impaired area in both older groups. Connectivity maps showed lower connectivity between the SFG and the left and right frontal poles as well as the medial frontal cortex in healthy older adults compared to young adults. Contrary to the expectation, healthy older adults did not differ from prodromal AD participants. Such findings suggest that, in healthy aging, no functional compensation occurs at the whole-brain level in terms of seeds that present massive changes at a voxel level (both on ILC and fALFF measures). The second seed that was chosen was the IFG-Orb, an area in which healthy older adults were less impaired (i.e., lower ILC values but similar fALFF to young adults). A different pattern emerged with this seed: healthy older adults did not differ from young adults but had more extended connectivity maps compared to prodromal AD participants. These results also explain why (Pistono et al., 2021a) found greater connectivity maps in older adults when the IFG was used as a seed. This area was only impaired in the prodromal AD group in this study. This indicates a crucial difference between healthy aging and prodromal AD. In fact, it would appear that healthy older adults do not have functional alterations at a seed-based level when the area analyzed is not, or only slightly impaired compared to young adults (i.e., IFG, IFG-Orb), while prodromal AD participants show decreased connectivity at a seed-based level as well. However, when healthy older adults are similar to participants with prodromal AD on both ILC and fALFF measures, their connectivity maps are also similar during seed-to-voxel analyses (i.e., SFG). Previous literature maintained that healthy aging is characterized by a decrease in connectivity between regions of the same network, accompanied by increases in the connectivity of these networks to regions of other networks (e.g., with the right hemisphere for the functional language network, Antonenko et al., 2013). Our results refine such findings by showing that this depends on the area of interest. In the aging brain, functional connectivity from the left IFG to the rest of the brain may in fact be characterized by increased connectivity with areas that are not part of the language network (Antonenko et al., 2013; Pistono et al., 2021a). However, this does not appear to be the case for other frontal areas of the language network, such as the SFG or the orbitofrontal gyrus. This finding also implies that the preservation of language performance in healthy aging relies on the functional compensation elicited by some but not all language-related areas.

Correlations highlighted differences between healthy aging and prodromal AD. In fact, in the group with prodromal AD, no correlations were found between measures of ILC or fALFF and language performance. In the healthy older group, higher ILC and fALFF values in frontal areas were positively correlated with fluency and naming tasks. Given that such local changes occur during the course of typical aging, the lack of significant correlations in the group with prodromal AD could be due to the fact that their language performance no longer relies on these local measures, but rather on the interaction between different language areas. Indeed, a previous study (Pistono et al., 2021b) showed that functional connectivity increases within prodromal AD participants’ language network, despite important gray matter loss. In particular, this study showed positive correlations, in the prodromal AD group, between the language production network (i.e., areas positively correlated with the left IFG) and language production tasks (i.e., phonemic fluency and sentence production). This suggests that this pattern of connectivity may compensate for the structural and functional impairment occurring at a more local level.

### Limitations

A predefined atlas was used to conduct these analyses, which might have influenced the results. However, the atlas we chose provides a good representation of the language function because it includes language-related areas that are not always part of other atlases, such as different sections of the left temporal lobe. We also decided to use an atlas that was functionally defined because it is more likely to represent brain regions actually involved in language processing rather than anatomical seeds (Muller and Meyer, 2014). Additionally, the sample size is rather small because of the homogeneity. We specifically recruited young adults with a comparable level of education to healthy older adults and prodromal AD participants, who were also monolingual.

### Implications and future directions

To our knowledge, this is the first study to investigate both the effect of healthy aging and prodromal AD on the functional language network and include a young adult group. These findings show that focusing on each language area is a sensitive method to examine the effects of typical aging. In fact, the inter-group differences noted were mostly due to the effect of aging, and not the effect of prodromal AD. They show a gradual decline from healthy aging to prodromal AD on these measures. The results also show that this method has an impact on seed-based analyses. Previous literature showed that healthy aging is characterized by functional compensation, in particular inter-network connections. This study suggests that these compensations may actually be limited, depending on the functional integrity of brain areas. Most importantly, the inclusion of a group of young adults highlighted that not all changes are due to prodromal AD, although language is usually described as preserved in typical aging. However, further work is required to replicate these findings, using other atlases and larger samples of participants to investigate language in the aging brain. Additionally, investigating functional lateralization and interhemispheric compensatory mechanisms (Zhao et al., 2020; Shahnazian et al., 2021), or using computational modeling (Schrimpf et al., 2021; Goldstein et al., 2022) approaches could help uncover the neural mechanisms involved in typical aging and prodromal AD.

## Data availability statement

The raw data supporting the conclusion of this article will be made available by the authors, without undue reservation.

## Ethics statement

The studies involving human participants were reviewed and approved by Toulouse University Hospital (CHU)–TellMA project grant number: 15050480. The patients/participants provided their written informed consent to participate in this study.

## Author contributions

MR: investigation, methodology, analyses, and writing–original draft. MJ: conceptualization, methodology, review and editing, project administration, and funding acquisition. LG: software, validation, and review and editing. PP: conceptualization, methodology, review and editing, and funding acquisition. JP: conceptualization, methodology, review and editing, and supervision. AP: conceptualization, methodology, investigation, writing–review and editing, and supervision. All authors contributed to the article and approved the submitted version.
