# Supplementary material for: The functional connectivity of language network across the life span: Disentangling the effects of typical aging from Alzheimer’s disease
Source: Front Aging Neurosci. 2022 Sep 23;14:959405. doi: 10.3389/fnagi.2022.959405 (PMC9537133; doi:10.3389/fnagi.2022.959405)

**Supplementary Material 1**

Descriptive values regarding ILC

|  |  | **Healthy older** | **MCI due to AD** | **Younger** |
| --- | --- | --- | --- | --- |
| LeftAngular | average | 0.627 | 0.621 | 0.628 |
|  | SD | 0.029 | 0.023 | 0.029 |
| LeftAntTemp |  | **Healthy older** | **MCI due to AD** | **Younger** |
|  | average | 0.601 | 0.601 | 0.617 |
|  | SD | 0.026 | 0.035 | 0.033 |
| LeftCerebellum |  | **Healthy older** | **MCI due to AD** | **Younger** |
|  | average | 0.585 | 0.578 | 0.596 |
|  | SD | 0.034 | 0.035 | 0.024 |
| LeftMFG |  | **Healthy older** | **MCI due to AD** | **Younger** |
|  | average | 0.587 | 0.583 | 0.599 |
|  | SD | 0.029 | 0.029 | 0.023 |
| LeftMidAntTemp |  | **Healthy older** | **MCI due to AD** | **Younger** |
|  | average | 0.617 | 0.615 | 0.619 |
|  | SD | 0.038 | 0.045 | 0.035 |
| LeftMidPostTemp |  | **Healthy older** | **MCI due to AD** | **Younger** |
|  | average | 0.649 | 0.630 | 0.644 |
|  | SD | 0.027 | 0.032 | 0.028 |
| LeftPostTemp |  | **Healthy older** | **MCI due to AD** | **Younger** |
|  | average | 0.642 | 0.644 | 0.644 |
|  | SD | 0.029 | 0.032 | 0.032 |
| RightCerebellum |  | **Healthy older** | **MCI due to AD** | **Younger** |
|  | average | 0.645 | 0.645 | 0.649 |
|  | SD | 0.034 | 0.031 | 0.034 |
| RightMidAntTemp |  | **Healthy older** | **MCI due to AD** | **Younger** |
|  | average | 0.630 | 0.621 | 0.634 |
|  | SD | 0.033 | 0.045 | 0.031 |
| RightMidPostTemp |  | **Healthy older** | **MCI due to AD** | **Younger** |
|  | average | 0.668 | 0.664 | 0.657 |
|  | SD | 0.030 | 0.033 | 0.027 |

**Supplementary Material 2**

Descriptive values regarding fALFF

|  |  | **Healthy older** | **MCI due to AD** | **Younger** |
| --- | --- | --- | --- | --- |
| LeftAngular | average | 0.740 | 0.736 | 0.742 |
|  | SD | 0.032 | 0.038 | 0.033 |
| LeftAntTemp |  | **Healthy older** | **MCI due to AD** | **Younger** |
|  | average | 0.745 | 0.742 | 0.758 |
|  | SD | 0.021 | 0.024 | 0.023 |
| LeftCerebellum |  | **Healthy older** | **MCI due to AD** | **Younger** |
|  | average | 0.730 | 0.734 | 0.740 |
|  | SD | 0.020 | 0.030 | 0.018 |
| LeftMFG |  | **Healthy older** | **MCI due to AD** | **Younger** |
|  | average | 0.709 | 0.711 | 0.720 |
|  | SD | 0.034 | 0.026 | 0.027 |
| LeftMidAntTemp |  | **Healthy older** | **MCI due to AD** | **Younger** |
|  | average | 0.750 | 0.744 | 0.758 |
|  | SD | 0.025 | 0.027 | 0.021 |
| LeftMidPostTemp |  | **Healthy older** | **MCI due to AD** | **Younger** |
|  | average | 0.771 | 0.761 | 0.772 |
|  | SD | 0.026 | 0.029 | 0.026 |
| LeftPostTemp |  | **Healthy older** | **MCI due to AD** | **Younger** |
|  | average | 0.763 | 0.765 | 0.763 |
|  | SD | 0.030 | 0.034 | 0.033 |
| RightCerebellum |  | **Healthy older** | **MCI due to AD** | **Younger** |
|  | average | 0.749 | 0.744 | 0.762 |
|  | SD | 0.027 | 0.030 | 0.022 |
| RightMidAntTemp |  | **Healthy older** | **MCI due to AD** | **Younger** |
|  | average | 0.750 | 0.746 | 0.760 |
|  | SD | 0.029 | 0.031 | 0.016 |
| RightMidPostTemp |  | **Healthy older** | **MCI due to AD** | **Younger** |
|  | average | 0.780 | 0.782 | 0.778 |
|  | SD | 0.029 | 0.035 | 0.024 |

**Supplementary Material 3**

Cluster maps for SFG in each group. Red color for clusters positively correlated to SFG activity; blue color for clusters negatively correlated to SFG activity. Cluster threshold p<.05, cluster size p-FDR corrected, Voxel threshold p<.001 p-uncorrected.


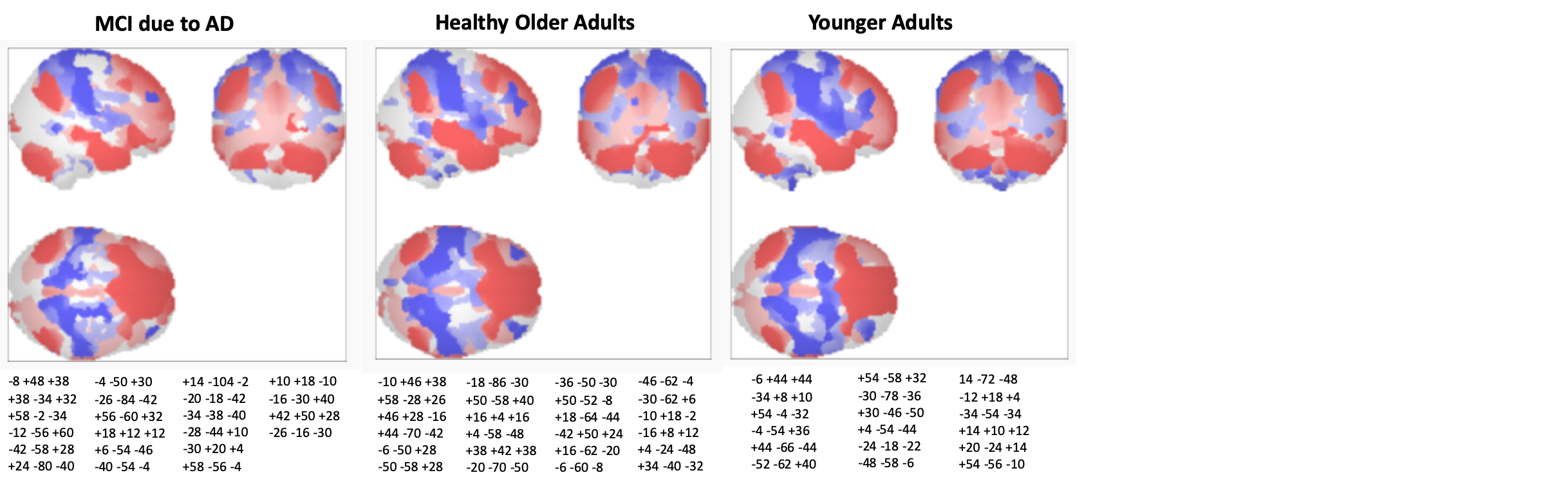


**Supplementary Material 4**

Cluster maps for IFG-Orb in each group. Red color for clusters positively correlated to IFG-Orb activity; blue color for clusters negatively correlated to IFG-Orb activity. Cluster threshold p<.05, cluster size p-FDR corrected, Voxel threshold p<.001 p-uncorrected.


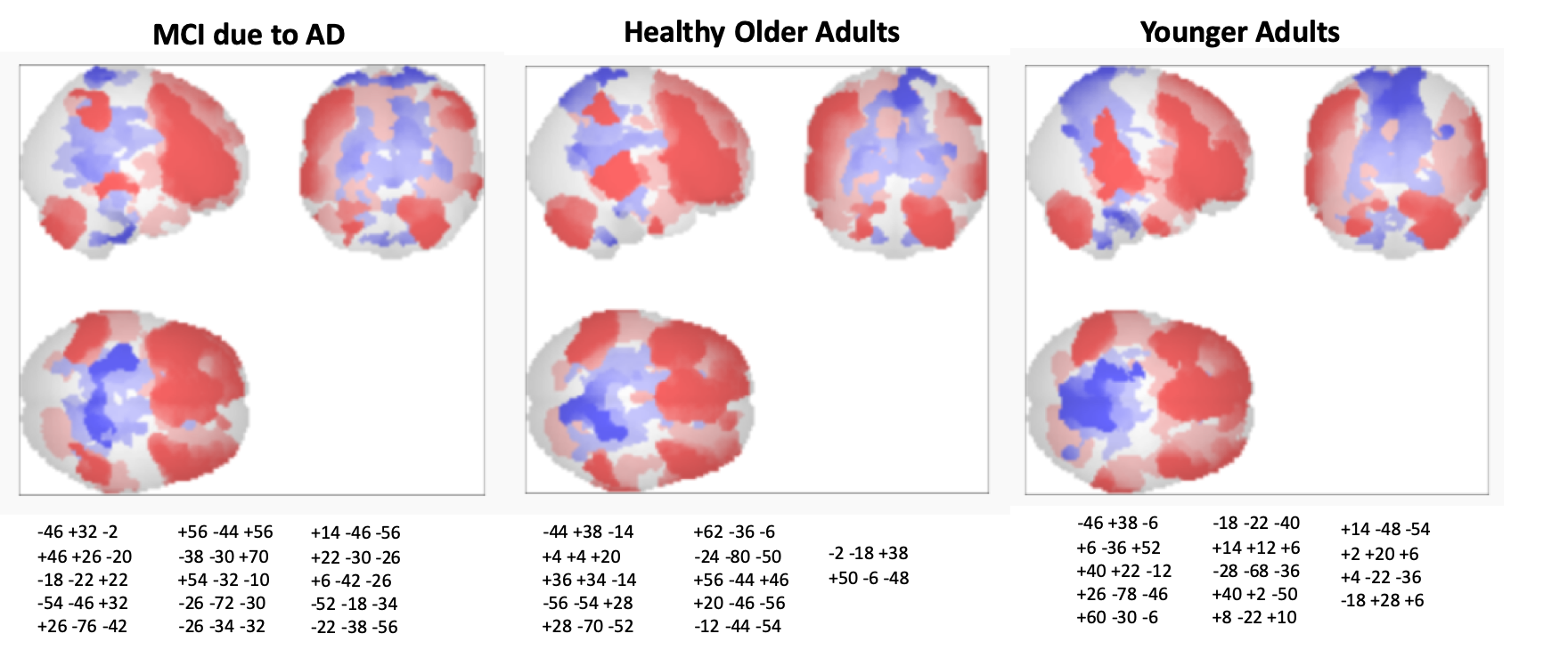

Supplement: Supplementary file 1 [file Data_Sheet_1.docx]
